# Biophysical processes of morphogenesis in lizard lungs

**DOI:** 10.1002/dvdy.70097

**Published:** 2025-11-14

**Authors:** Kaleb Hill, Aaron H. Griffing, Michael A. Palmer, Bezia Lemma, Aria S. Lupo, Tony Gamble, Natalia A. Shylo, Andrej Košmrlj, Paul A. Trainor, Celeste M. Nelson

**Affiliations:** ^1^ Department of Chemical & Biological Engineering Princeton University Princeton New Jersey USA; ^2^ Department of Molecular Biology Princeton University Princeton New Jersey USA; ^3^ Milwaukee Public Museum Milwaukee Wisconsin USA; ^4^ Marine Biological Laboratory Woods Hole Massachusetts USA; ^5^ Department of Ecology & Evolutionary Biology Princeton University Princeton New Jersey USA; ^6^ Department of Biological Sciences Marquette University Milwaukee Wisconsin USA; ^7^ Bell Museum of Natural History University of Minnesota St. Paul Minnesota USA; ^8^ Department of Biological & Biomedical Sciences Rowan University Glassboro New Jersey USA; ^9^ Department of Mechanical and Aerospace Engineering Princeton University Princeton New Jersey USA; ^10^ Stowers Institute for Medical Research Kansas City Missouri USA; ^11^ Department of Physiology and Cell Biology University of Kansas Medical Center Kansas City Kansas USA

**Keywords:** mechanical stress, tissue morphodynamics, tissue folding

## Abstract

**Background:**

The lungs of squamate reptiles (lizards and snakes) are highly diverse, exhibiting single chambers, multiple chambers, transitional forms with two to three chambers, along with a suite of other anatomical features, including finger‐like epithelial projections into the body cavity known as diverticulae. During embryonic development of the simple, sac‐like lungs of anoles, the epithelium is pushed through the openings of a pulmonary smooth muscle mesh by the forces of luminal fluid pressure. This process of stress ball morphogenesis generates the faveolar epithelium typical of squamate lungs.

**Results:**

Here, we compared embryonic lung development in brown anoles, leopard geckos, and veiled chameleons to determine if stress ball morphogenesis is conserved across squamates and to understand the physical processes that generate transitional‐chambered lungs with diverticulae. We found that epithelial protrusion through the holes in a pulmonary smooth muscle mesh is conserved across squamates. Surprisingly, however, we found that luminal inflation is not conserved. Instead, experimental and computational evidence suggests that leopard geckos and veiled chameleons may generate their faveolae via epithelial folding downstream of epithelial proliferation. Our data also suggest that the transitional chambers and diverticulae of veiled chameleon lungs may develop via apical constriction, a process known to be crucial for airway branching in the bird lung.

**Conclusions:**

Distinct morphogenetic mechanisms generate epithelial diversity in squamate lungs, which may underpin their species‐specific physiological and ecological adaptations.

## 1 INTRODUCTION

The vertebrate lung exhibits strikingly diverse epithelial architectures, including the tree‐like airways of mammals, the looped airways of birds, and the corrugated, faveolar sacs of amphibians and non‐avian reptiles.^1^, ^2^ The latter span the extremes of epithelial complexity. More complex forms include the multi‐chambered and multi‐lobed lungs of turtles,^3^, ^4^ the multi‐chambered lungs of crocodilians,^3^, ^5^ and the multi‐chambered sac‐like lungs of some lizards and snakes.^3^, ^4^ Less complex forms include the single‐chambered sac‐like lungs of most lizards and tuatara.^3^, ^6^ To achieve this variation in epithelial morphology, different vertebrate lineages appear to use distinct morphogenetic mechanisms during early lung development.^7^ The epithelium of the mammalian lung forms branches in response to mechanical constraints from patterns of smooth muscle differentiation.^8^, ^9^, ^10^ The epithelium of the bird lung folds via apical constriction and later fuses to generate the circuit for unidirectional airflow.^11^, ^12^, ^13^, ^14^ The epithelium of the lungs of lizards and clawed frogs expands through openings in a smooth muscle mesh, apparently in response to inflation of the lumen with fluid.^15^, ^16^ This process, known as stress ball morphogenesis,^16^ is required for lung development in anole lizards, but it remains unclear whether stress ball morphogenesis is conserved in other reptiles.

**FIGURE 1:**
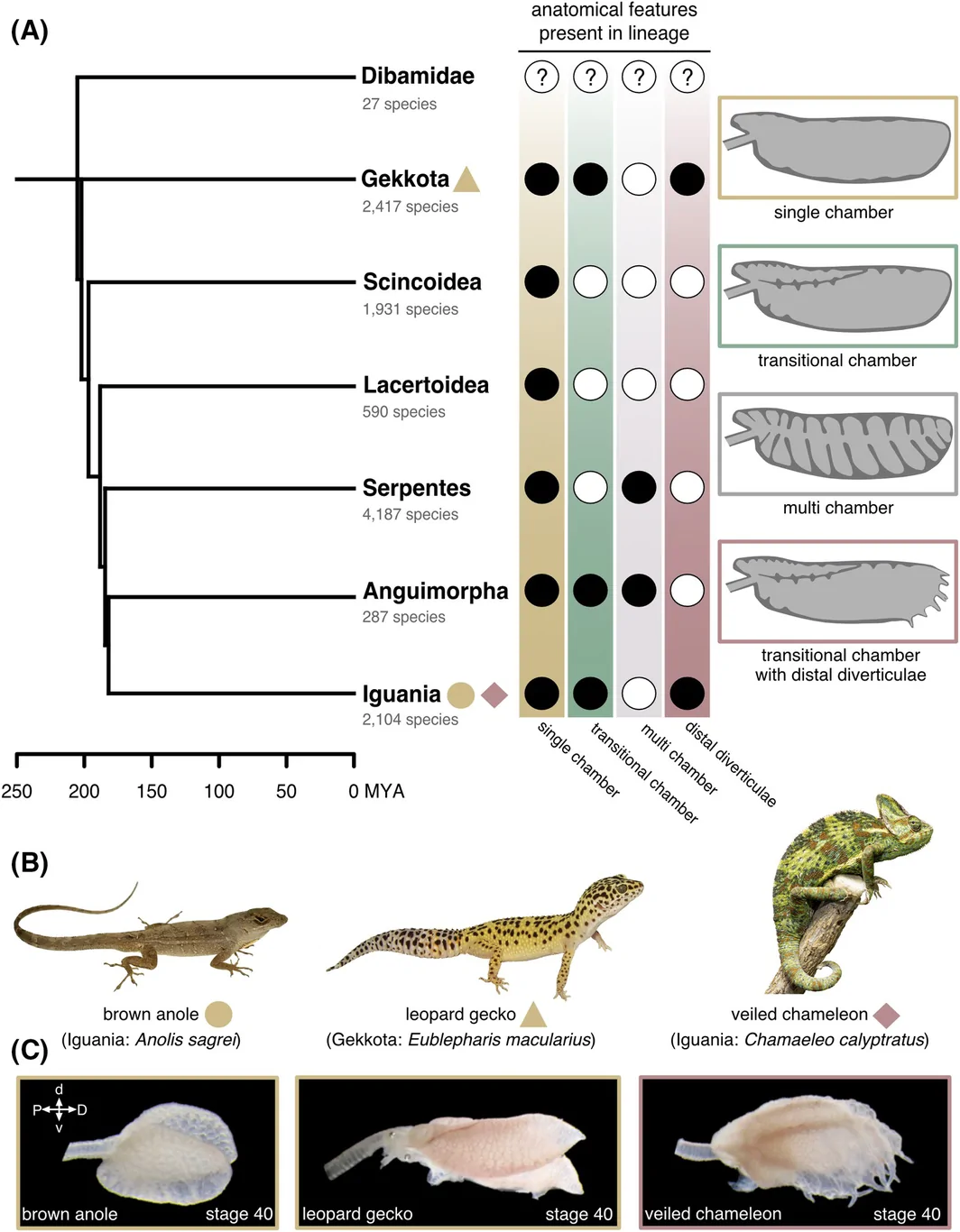
Lung diversity in squamate reptiles. (A) Major lineages of squamate reptiles in a phylogenetic context and anatomical features present in each lineage. Black and white circles correspond to presence and absence of anatomical features in the lineage, respectively. Anatomical data from Perry.^3^ Phylogeny modified from Zheng and Wiens.^28^ (**B**) Photographs of squamate species investigated in this study. (**C**) Lateral view of near‐hatching embryonic lungs of species investigated in this study. D, distal; d, dorsal; MYA, millions of years ago; P, proximal; v, ventral.

Squamates encompass a lineage of more than 12,000 described species of lizards and snakes with diverse morphologies and physiologies that have facilitated their ecological radiation and adaptation.^17^ Their lungs are similarly diverse and can be categorized into three chamber types: single chamber, transitional chamber, or multi‐chamber. All major squamate lineages have species that exhibit single‐chambered lungs, which are simple undivided sacs (Figure 1A).^3^ Several lineages within gekkotan, anguimorph, and iguanian lizards exhibit transitional‐chambered lungs, which have two or three chambers separated by internal longitudinal septa (Figure 1A).^3^, ^18^, ^19^ Some snakes and anguimorph lizards exhibit multi‐chambered lungs with cartilage‐reinforced bronchi (Figure 1A).^3^, ^20^, ^21^, ^22^ In addition, three lineages of squamates exhibit finger‐like outpouchings of epithelial tissue known as diverticulae: *Uroplatus* geckos,^23^, ^24^ *Polychrus* lizards,^25^ and chameleons (Figure 1A).^18^ As compared with single‐chambered lungs, transitional and multi‐chambered lungs are thought to have increased surface area for gas exchange,^26^, ^27^ an ability to store air,^3^ and a morphology that permits unidirectional airflow.^4^, ^19^ The function of diverticulae remains unknown; however, these epithelial outpouchings may increase lung volume in arboreal lineages that have compressed torsos.^18^, ^26^ The morphogenetic processes that build these more complex epithelial structures are also unknown.

Here, we investigated embryonic lung development in three species of squamates to identify the extent to which stress ball morphogenesis is conserved and to uncover the physical processes that generate transitional chambers and diverticulae. We found that meshes of pulmonary smooth muscle are present in each lineage we studied, suggesting that this tissue structure may be conserved across squamates. However, different species generate faveolae via epithelial growth through the mesh, as opposed to luminal pressure pushing the epithelium through the mesh. Our data also suggest that the individual chambers and diverticulae of the chameleon lung may initiate via apical constriction, the process that induces branch formation in the avian lung. Taken together, our results suggest that a variety of biophysical processes are used to generate the diverse epithelial morphologies observed in squamate lungs.

## 2 RESULTS

We focused our analysis on three model squamate species: the brown anole (*Anolis sagrei*), leopard gecko (*Eublepharis macularius*), and veiled chameleon (*Chamaeleo calyptratus*; Figure 1B). Both brown anoles and veiled chameleons are iguanian lizards, while leopard geckos are gekkotan lizards (Figure 1A). Because gekkotan lizards, with the possible exception of dibamids, are the sister lineage to all remaining squamates, our taxon sampling scheme spans almost the entire evolutionary history of Squamata.^28^ Before hatching, the lungs of squamates are morphologically similar to adult lungs and contain faveolar epithelium.^29^ Both brown anoles and leopard geckos have single‐chambered lungs with slight proximal subchambers (Figure 1C).^3^, ^16^, ^30^ Veiled chameleons have lungs with three main chambers and distally situated diverticulae.^18^

**FIGURE 2:**
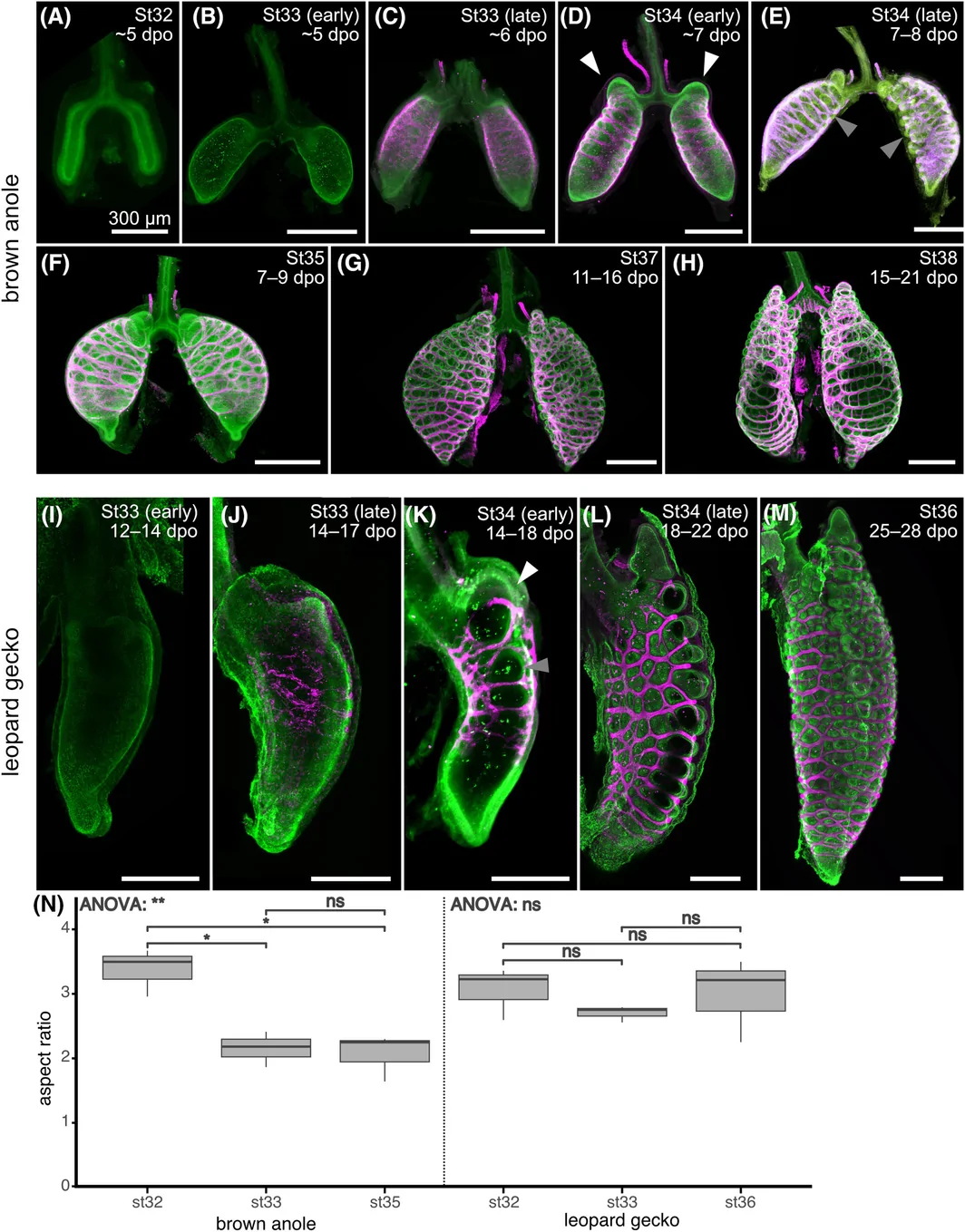
Developmental staging of brown anole and leopard gecko lungs. Images show staining for F‐actin (green; A) or immunofluorescence for E‐cadherin (green; B–M) and alpha smooth muscle actin (αSMA; magenta). dpo, days postoviposition; St, developmental stage. Scale bars, 300 μm. (N) Aspect ratios of developing lungs of brown anoles and leopard geckos. White arrows indicate proximal sub‐chamber of both species. Gray arrows indicate epithelium pushing through the smooth muscle mesh. Asterisks and ns denote significant and non‐significant differences, respectively, in one‐way ANOVAs. Brown anole all stages ANOVA (*p* = .006), st32 vs. st33 (*p* = .010), st32 vs. st35 (*p* = .012), st33 vs. st35 (*p* = .751). Leopard gecko all stages ANOVA (*p* = .612), st32 vs. st33 (*p* = .219), st32 vs. st35 (*p* = 878), st33 vs. st35 (*p* = .498).

Development *in ovo* is variable in squamates and sensitive to temperature. When incubated at 27 ± 1°C, brown anoles hatch after ~27 days,^31^ leopard geckos hatch after ~52 days,^32^ and veiled chameleons hatch after ~200 days.^33^ We therefore staged embryos using morphological criteria.^31^, ^32^, ^34^, ^35^, ^36^ Lungs were dissected from embryos between stages 32 and 38 and labeled for markers of epithelium and smooth muscle using immunofluorescence. At stage 32, the brown anole lung begins as a simple wishbone‐shaped epithelium without pulmonary smooth muscle (Figure 2A). During stage 33, the lung begins to inflate and, soon after, smooth muscle begins differentiating and surrounding the epithelium (Figure 2B,C). At stage 34, the pulmonary smooth muscle forms into a meshwork and the lumen of the lung further inflates with fluid, decreasing the aspect ratio of each simple sac (Figure 2D,E). A proximal subchamber is visible during this stage (Figure 2D). From stage 34 onward, inflation of the lumen pushes the epithelium through the holes in the smooth muscle mesh (Figure 2D–H), generating the initial morphology of the faveolae.^16^

We found that the development of the lung of the leopard gecko follows a slightly different series of morphogenetic events. At stage 33, the epithelium is a simple wishbone‐shaped sac, similarly absent of pulmonary smooth muscle (Figure 2I). Between stages 33 and 34, pulmonary smooth muscle differentiates and forms into a meshwork (Figure 2J,K). During late stage 34, the epithelium has begun to protrude through holes in the pulmonary smooth muscle mesh (Figure 2L), and a proximal subchamber is similarly structured. By stage 36, the mesh has become more refined and the initial morphology of the faveolae is established (Figure 2M). However, we noticed that the epithelium of the leopard gecko lung does not appear to inflate during these time points. To confirm this qualitative observation, we measured the aspect ratios of brown anole and leopard gecko lungs before the appearance of smooth muscle and after the deformation of the epithelium. These measurements confirmed that the lung of the brown anole inflates during these stages (i.e., decreases in aspect ratio between stages) but the lung of the leopard gecko does not (Figure 2N; Table S1).

**FIGURE 3:**
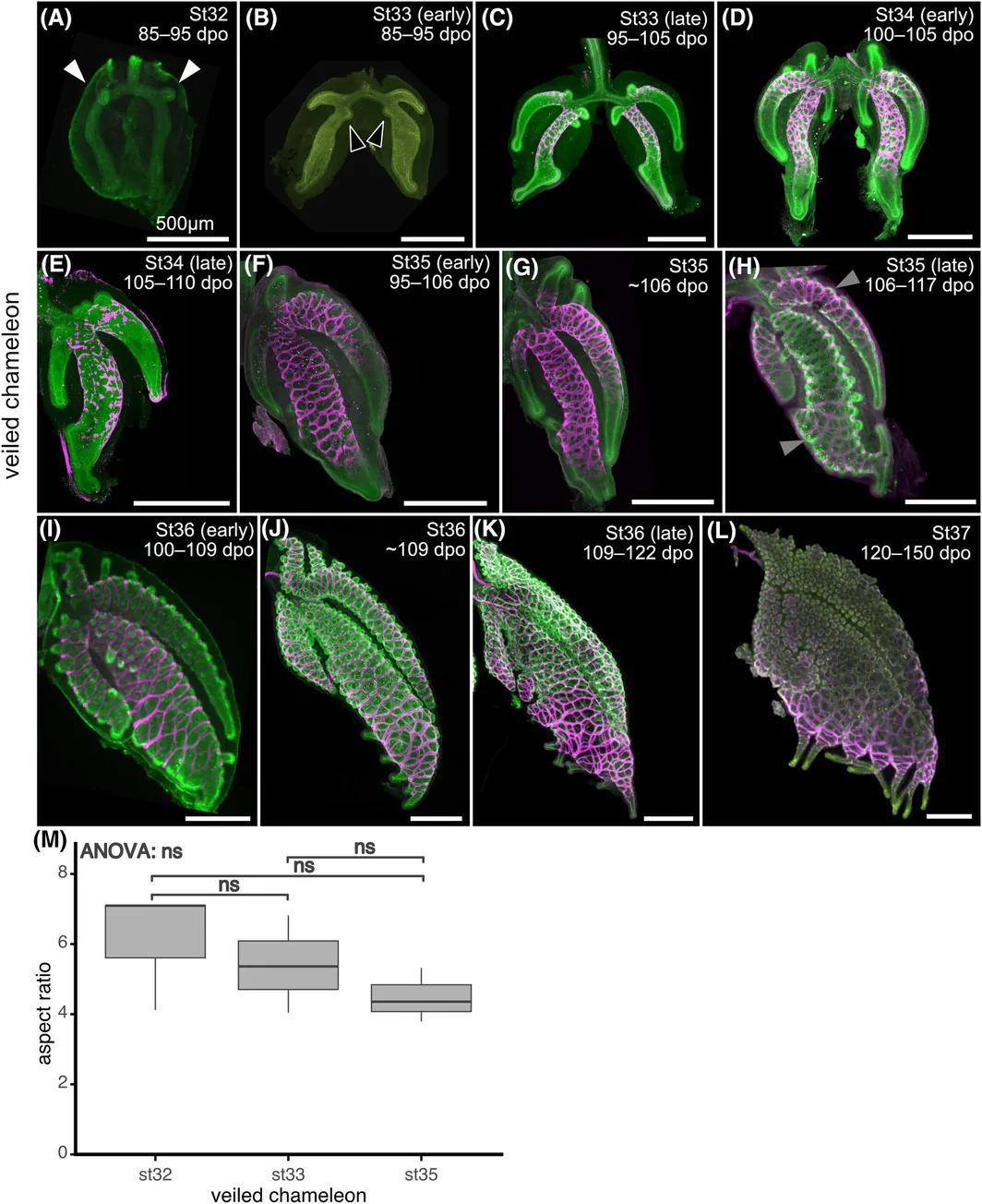
Developmental staging of veiled chameleon lungs. Images show immunofluorescence for αSMA (magenta) and either epithelial cytokeratin (A, I, L) or E‐cadherin (B–H, J–K). dpo, days postoviposition; St, developmental stage, Scale bars, 500 μm. (M) Aspect ratios of developing lungs of veiled chameleons. White and black arrows indicate emergence of the ventral subbronchus and dorsal subbronchus, respectively. Gray arrows indicate epithelium pushing through the smooth muscle mesh. ns denotes non‐significant differences in one‐way ANOVAs (*p* = .397), st32 vs. st33 (*p* = .612), st32 vs. st35 (*p* = .212), st33 vs. st35 (*p* = .373).

We found that the development of the transitional‐chambered lung of the veiled chameleon is distinct from that of both the brown anole and leopard gecko. At stage 32, the veiled chameleon lung exhibits a wish‐bone‐shaped epithelium with proximally situated buds that will become the ventral subbronchi (Figure 3A), with no visible pulmonary smooth muscle. At early stage 33, the ventral subbronchus has elongated and the emerging buds of the dorsal subbronchus are visible (Figure 3B). At late stage 33, pulmonary smooth muscle has begun to differentiate around the epithelium of the main bronchus and the most proximal portion of the ventral subbronchus (Figure 3C). At this stage, an additional sub‐chamber is visible proximally to the ventral subbronchus (Figure 3C). At stage 34, both subbronchi and the proximal sub‐chamber have elongated (Figure 3D,E). Smooth muscle has begun to differentiate distally around the main bronchus and wrap around the ventral subbronchus (Figure 3D,E). At stage 35, the subbronchi have elongated further while smooth muscle has continued to wrap in a distal direction around the subbronchi and main bronchus (Figure 3F,G). At late stage 35, smooth muscle has formed a meshwork around most of the epithelium, which has started to protrude through the holes in the mesh (Figure 3H). From stage 36 onward, the epithelial protrusions become more prominent and the lung appears to inflate (Figure 3I–L). Nonetheless, the aspect ratio of the veiled chameleon lung remains constant during the stages of development in which the epithelium pushes through the smooth muscle mesh (Figure 3M; Table S1).

**FIGURE 4:**
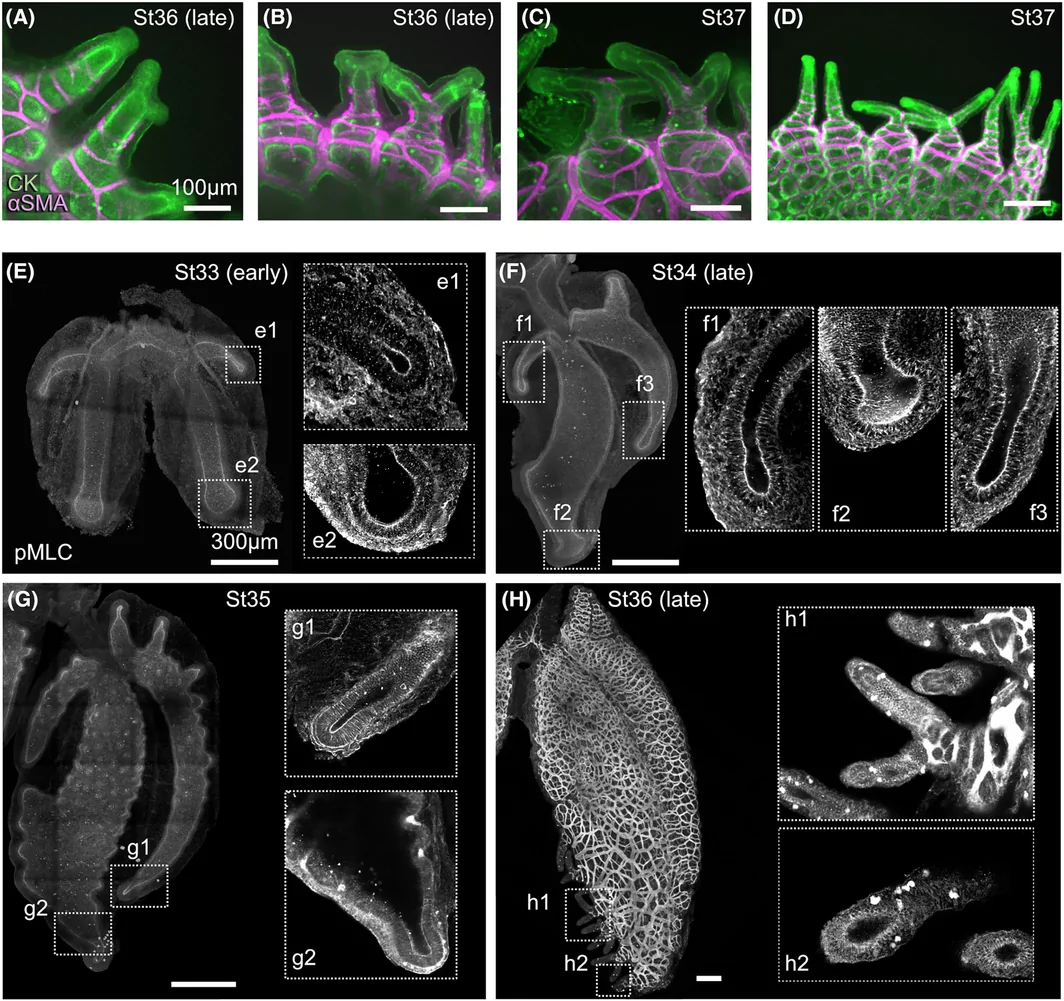
Analysis of smooth muscle coverage and apical constriction in diverticulae and subbronchi of veiled chameleon lungs. (A–D) Images showing immunofluorescence for cytokeratin (CK) and αSMA in the veiled chameleon lung diverticulae. (E–H) Images showing immunofluorescence for phosphorylated myosin light chain (pMLC). Scale bars (A–D), 100 μm. Scale bars (E–H), 300 μm.

In addition to being transitional‐chambered, the veiled chameleon lung differs from those of the brown anole and leopard gecko by having diverticulae, which first appear at stage 36 (Figure 4A). At this stage, smooth muscle wraps around the base of each diverticulum (Figure 4A). At late stage 36, some, but not all, diverticulae have begun to bifurcate (Figure 4B). By stage 37, the diverticulae are long, finger‐like structures that resemble the diverticulae within the lungs of adult veiled chameleons (Figure 4C,D). These epithelial structures are more similar in morphology to the branched airways of the mammalian and avian lungs than to faveolae. In the mouse lung, epithelial branches are sculpted by patterns of smooth muscle differentiation,^8^, ^9^, ^10^ which specifies sites of bifurcation prior to changes in epithelial shape. The epithelium of the diverticulae, however, appears to bifurcate before smooth muscle differentiates at the bifurcation site (Figure 4A,B). In the bird lung, epithelial branches form by active actomyosin‐induced contraction of the epithelium itself, in a process known as apical constriction.^11^, ^12^ To determine whether apical constriction might play a role in the morphogenesis of the veiled chameleon lung, we conducted immunofluorescence analysis for phosphorylated myosin light chain (pMLC). We observed increased staining intensity for pMLC at the apical surface of the epithelium at the distal tips of the subbronchi and main bronchi from stages 33 to 35 (Figure 4E–G). We also observed a striking enrichment in pMLC at the apical surface of the epithelium in diverticulae (Figure 4H). These data suggest that apical constriction may play a role in the initiation of the epithelial folding that constructs both the subbronchi as well as diverticulae in the veiled chameleon lung.

**FIGURE 5:**
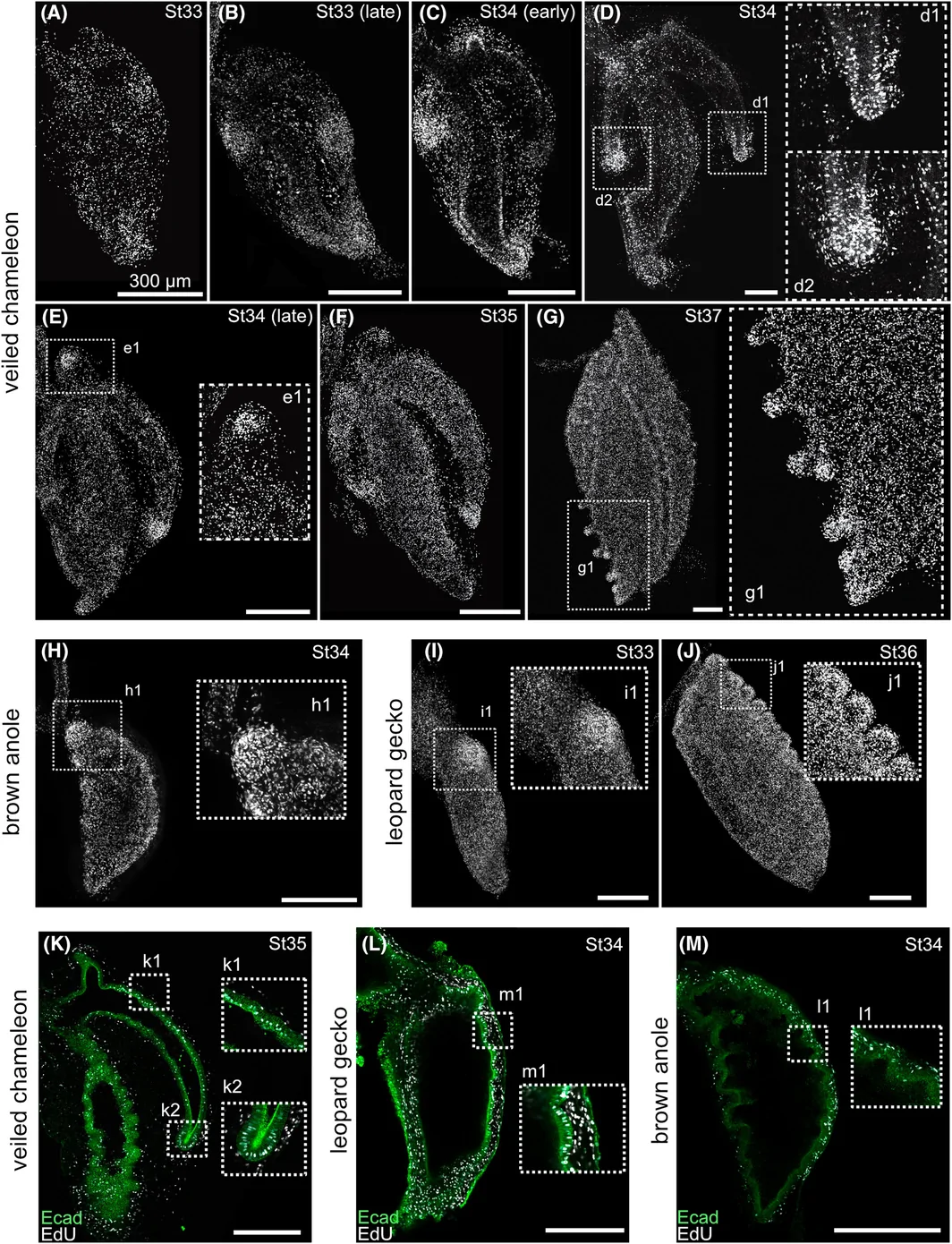
Patterns of cell proliferation in the developing lungs of lizards. (A–G) Images showing EdU incorporation in the veiled chameleon lung. (H) Image showing EdU incorporation in the brown anole lung. (I–J) Images showing EdU incorporation in the leopard gecko lung. (K–M) Z‐slice images showing immunofluorescence for E‐cadherin (green) and fluorescent EdU incorporation (cyan) in the veiled chameleon (K), leopard gecko (L), and brown anole (M). Scale bars, 300 μm.

After the avian airway epithelium changes shape to form a branch, cells at the tip begin to proliferate at a higher rate than the adjacent epithelium, which promotes branch elongation.^11^, ^13^ To determine whether a similar spatial pattern of proliferation accompanies morphogenesis of the subbronchi and diverticulae in the chameleon lung, we isolated lungs at different stages of development and monitored incorporation of the thymidine analog, EdU. From late stage 33 onward, EdU incorporation was substantially higher in epithelial cells located at the growing tips of subbronchi and the proximal sub‐chamber than adjacent regions of the lung (Figure 5A–F). Similarly, at stage 37, EdU incorporation was higher in the tips of the diverticulae (Figure 5G). We also found high EdU incorporation in the developing proximal sub‐chambers of brown anole (Figure 5H) and leopard gecko (Figure 5I). In the faveolae of both the veiled chameleon and leopard gecko, we observed EdU incorporation in the epithelium as well as the mesenchyme (Figure 5J–L). In contrast, we observed EdU incorporation primarily in the mesenchyme surrounding faveolar epithelium of the brown anole (Figure 5M), consistent with previous observations.^16^ Overall, our data suggest that the faveolar epithelia of leopard geckos and veiled chameleons are highly proliferative. In addition, veiled chameleons exhibit high levels of proliferation in the elongating epithelium of the subbronchi and diverticulae.

**FIGURE 6:**
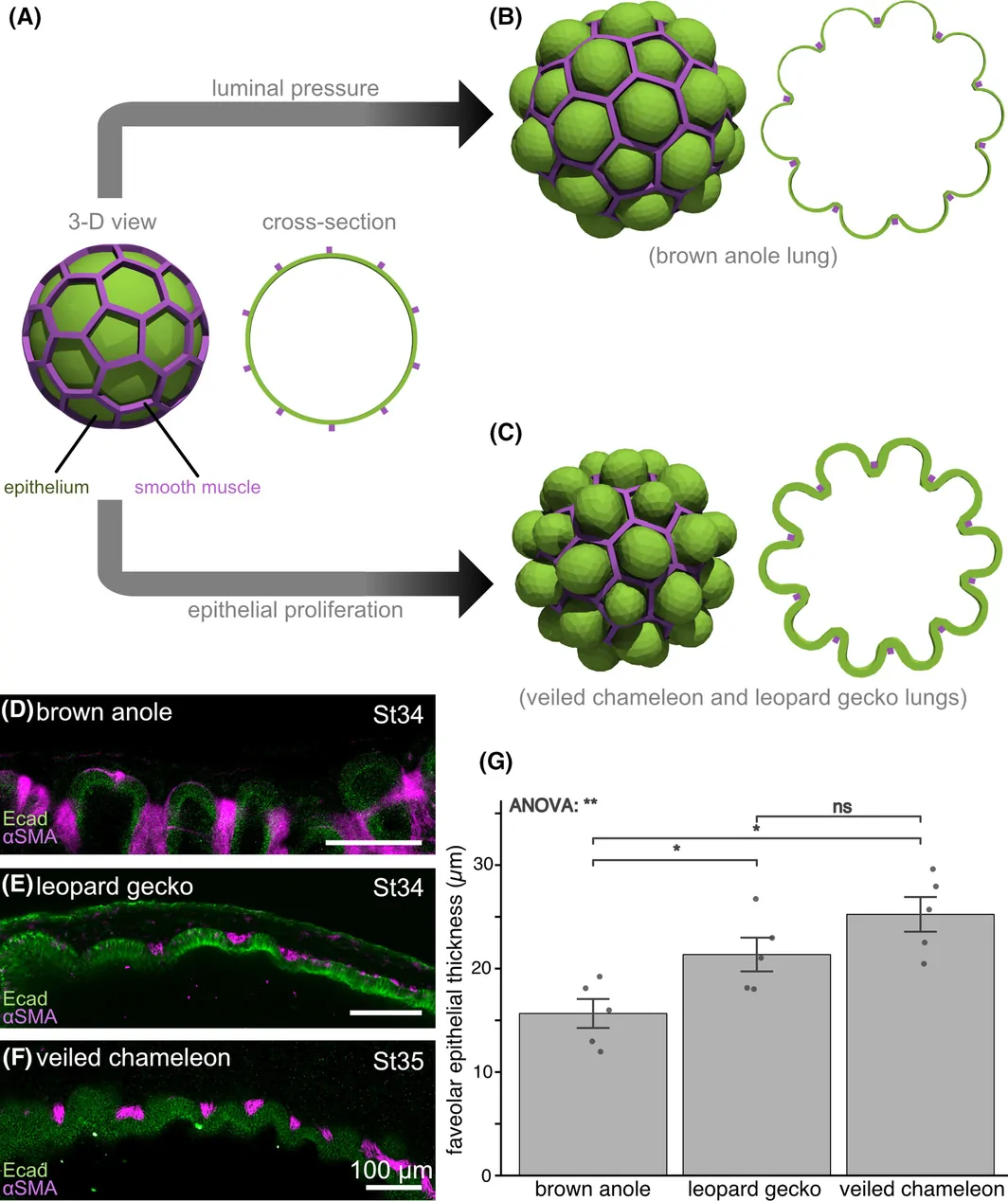
Computational simulations of epithelial deformations downstream of luminal pressure or epithelial growth predict epithelial thickness. (A) The model begins as a smooth shell of epithelium (green) contained within a smooth muscle mesh (magenta.) The model experiences either increased luminal pressure (B) or epithelial growth (C). (D–F) Z‐slice images showing immunofluorescence for E‐cadherin (green) and αSMA (magenta) in the developing faveolae of the brown anole (D), leopard gecko (E), and veiled chameleon (F). Scale bars, 100 μm. (G) Graph showing epithelial thickness during faveolar morphogenesis. Asterisks and ns denote significant and non‐significant differences, respectively, in one‐way ANOVA. All comparisons ANOVA (*p* = .009), brown anole vs. leopard gecko (*p* = .029), brown anole vs. veiled chameleon (*p* = .023), leopard gecko vs. veiled chameleon (*p* = .136).

During development of the anole lung, the volume of the fluid within the lumen of the organ increases between stages 32 and 33 (a span of 24 h) and the resulting pressure is sufficient to expand the epithelium and push it through the holes in the smooth muscle mesh.^16^ That neither the leopard gecko (Figure 2N) nor the veiled chameleon (Figure 3M) show an increase in aspect ratio during faveolar morphogenesis suggests that this process might occur independently of luminal pressurization in these species. Furthermore, the leopard gecko and veiled chameleon exhibit substantial proliferation in the regions of epithelium that fold into faveolae (Figure 5K,L). We hypothesized that the growth from this elevated proliferation might be sufficient to deform the epithelium through the holes in the smooth muscle mesh. The process of faveolar morphogenesis is relatively slow in these species (~3–4 days in leopard gecko; ~10 days in veiled chameleons), which unfortunately precludes experimental manipulation in culture. We therefore tested this hypothesis computationally using the finite element method. Specifically, we modeled the epithelium as a smooth, proliferative surface surrounded by a rigid lattice that matched the relative geometry of the squamate lungs (Figure 6A). Increasing pressure of the lumen pushes the simulated epithelium through the holes in the smooth muscle mesh (Figure 6B), as observed previously.^16^ Intriguingly, we found that in the absence of pressurization, growth of the simulated epithelium is also sufficient to expand this tissue through the mesh and generate a corrugated faveolar‐like surface (Figure 6C). Importantly, our computational model predicts that this proliferation‐driven faveolar folding would also occur independently of epithelial thinning. To test this prediction experimentally, we measured the relative thickness of the epithelium during faveolar morphogenesis in all three species (Figure 6D–F). As predicted, the faveolar epithelium of the brown anole was significantly thinner than that of both the leopard gecko and veiled chameleon (Figure 6G), which were similar in thickness to each other. These data suggest that there are at least two physical mechanisms to fold the epithelium into faveolae: pressure‐driven pushing and proliferation‐driven expansion.

## 3 DISCUSSION

The lungs of brown anoles, leopard geckos, and veiled chameleons all start as wishbone‐shaped epithelial tubes devoid of pulmonary smooth muscle. Around late stage 33, smooth muscle differentiates and subsequently organizes into a mesh. Based on our taxon sampling, differentiation of smooth muscle and remodeling into a mesh appear to be conserved across squamates. Consistent with our hypothesis, it was recently reported that the epithelium folds through holes lined by bundles of smooth muscle in the lung of the corn snake (*Pantherophis guttatus*).^37^ It remains unclear what promotes pulmonary smooth muscle differentiation in squamates. Shh signaling is required for pulmonary smooth muscle differentiation in the murine embryonic lung.^8^, ^9^ Similarly, blocking the Shh pathway with cyclopamine prevents smooth muscle differentiation in brown anoles, while activating Shh signaling with smoothened agonist results in ectopic smooth muscle.^16^ Additional work is needed to characterize the signaling pathways that regulate smooth muscle differentiation in squamates, broadly.

The subbronchi of chameleon lungs branch off from the main bronchus and elongate. After subbronchial elongation and faveolar morphogenesis, the diverticulae develop. These structures form in the absence of an obvious smooth muscle template, but show elevated pMLC at the apical surface of the epithelium, suggesting they may initiate via apical constriction, which folds the epithelium of the bird lung.^11^ Our observations suggest that apical constriction might be conserved across avian and non‐avian reptiles as a mechanism for pulmonary outgrowth and elongation. The molecular signals that induce the formation of subbronchi and diverticulae remain to be uncovered.

Fluid pressure serves as the physical force responsible for deforming the epithelium into faveolae in the anole lung.^16^ This inflation‐driven process does not appear to occur during faveolar morphogenesis in either the leopard gecko or veiled chameleon. Instead, the actively growing epithelium seems to push itself through the holes in the smooth muscle mesh, similar to the growth‐driven expansion of alveoli through mesenchymal rings in the mouse lung.^38^, ^39^, ^40^ This difference in physical mechanism may be the result of developmental timing. Brown anoles develop more rapidly than leopard geckos and veiled chameleons. Increasing luminal pressure in the anole lung generates faveolae quickly, instead of the multi‐day proliferative process observed in geckos and chameleons. Consistent with this hypothesis, opportunistic embryonic sampling of the lacertoid Italian wall lizard (*Podarcis siculus*) revealed a decrease in aspect ratio during the period in which faveolae form (Figure S1), suggesting pressure‐driven expansion in this faster developing species. It is also possible that developmental mechanisms are related to pulmonary physiology and/or specialization in the adult. Sampling additional squamate species,^29^ including those that exhibit diversity of physiology in the adult and developmental lengths *in ovo*, may help to uncover additional developmental and mechanical constraints on epithelial morphogenesis. Furthermore, studying additional tissues in the developing squamate lung, such as the vascular endothelium, mesothelium, and nerves, may provide additional insights into tissue interactions that facilitate biophysical mechanisms, morphological diversification, ecological radiation, and adaptation.

## 4 EXPERIMENTAL PROCEDURES

### 4.1 Lizard husbandry, egg collection, and dissection

We obtained eggs from a colony of wild‐caught brown anoles (*Anolis sagrei*) housed at Princeton University (Princeton, NJ). We performed husbandry, breeding, and maintenance as previously described^16^ and all animal procedures were approved by the Princeton University Institutional Animal Care and Use Committee (IACUC; protocol 2104). Briefly, we established ratios of one male to three females per enclosure, maintained at ambient temperature (23.9–29.4°C) and humidity (>80%). We obtained eggs from a colony of captive‐born leopard geckos (*Eublepharis macularius*) housed at Marquette University (Milwaukee, WI). We performed husbandry, breeding, and maintenance as previously described^41^ and all animal procedures were approved by the Marquette University IACUC (protocols MU‐4192 and MU‐4241). Briefly, we established ratios of either one male to one female or one male to two females per enclosure. Further, we maintained ambient temperature (24.0–27.8°C), a localized “warm spot” in each enclosure (28–31°C), and ambient humidity (>75%). We obtained eggs from a colony of captive‐born veiled chameleons (*Chamaeleo calyptratus*) housed at the Stowers Institute for Medical Research (Kansas City, MO). We performed husbandry, breeding, and maintenance as previously described^33^, ^42^, ^43^ and all animal procedures were approved by the Stowers Institute for Medical Research IACUC (protocol 2023‐160). Briefly, we housed chameleons individually and introduced females to male enclosures for <8 h for breeding, maintained at ambient temperature (21–24°C) and humidity (~50%). Finally, we opportunistically obtained eggs from a colony of wild‐caught Italian wall lizards (*Podarcis siculus*) housed at Princeton University. We performed husbandry, breeding, and maintenance similarly to brown anoles^16^ and all animal procedures were approved by the Princeton University IACUC (protocols 3154 and 3155).

Prior to dissection, we incubated eggs of all four species at Princeton University in a Vivarium Electronics VE‐200 incubator in damp vermiculite at 26.7°C. We removed embryos by submerging eggs in phosphate‐buffered saline (PBS), piercing the eggshell using #5 watchmaker's forceps (Fine Science Tools), and gently removing the eggshell and extraembryonic membranes.^44^, ^45^ We staged embryos using morphological criteria^31^, ^32^, ^34^, ^35^, ^36^ and selected embryos from stages 32 to 38. We dissected out lizard lungs by making three cuts using watchmaker's forceps: one postcranial cut, one anterior to the pelvis, and finally removing the dorsal body wall and spine (Figure S2). After removing the dorsum of the embryo, we used forceps to gently remove the viscera and adjacent organs until we isolated the lung and trachea (Figure S2).

### 4.2 Immunofluorescence

We fixed lungs in 4% paraformaldehyde in PBS for 15–30 min at room temperature. We then washed samples four times in 0.1% Triton X‐100 (Sigma‐Aldrich) in tris‐buffered saline (TBST) for 30 min each and then blocked for 1 h at room temperature in a solution of 5% goat serum (Sigma‐Aldrich) and 0.1% bovine serum albumin (Sigma‐Aldrich) in 0.1% TBST. We placed samples in solutions of primary antibody targeting epithelial cytokeratins (CK; 1:100; DSHB, TROMA‐I; 1:400; Dako, Z0622), E‐cadherin (Ecad; 1:200; Invitrogen, 13‐1900), α‐smooth muscle actin (αSMA; 1:200; Abcam, ab5694; 1:400; Sigma‐Aldrich, 1A4), and/or phosphorylated myosin light chain 2 (pMLC; 1:50; Cell Signaling Technology, 3675S) for 48 h at 4°C. We then washed samples in TBST four times for 30 min each and subsequently placed samples in a solution of secondary antibody (1:400; Invitrogen) for 48 h at 4°C. We then dehydrated samples with serial dilutions of either isopropanol or methanol in PBS (25%, 50%, 75%, and 100%). Finally, we cleared samples using Murray's clear (1:2 benzyl alcohol to benzyl benzoate; Sigma‐Aldrich) and mounted samples in imaging chambers composed of nylon washers affixed to glass coverslips and filled with Murray's clear.

### 4.3 EdU analysis

We visualized cell proliferation using a fluorescent assay for the thymidine analog 5‐ethynyl‐2′‐deoxyuridine (EdU; Invitrogen C10638). We placed dissected lung explants on a semipermeable membrane (8‐μm pore; Whatman) floating on Dulbecco's modified Eagle's medium (DMEM)/F‐12 medium (Hyclone) supplemented with 5% fetal bovine serum (FBS; Gemini Bioproducts). We cultured samples at 37°C for 30 min and then pulsed with EdU Click‐iT reaction for an additional 30 min. Finally, we fixed lungs in 4% paraformaldehyde in PBS for 15 min at room temperature and proceeded to perform immunofluorescence in tandem with EdU (see Section 4.2).

### 4.4 Fluorescence imaging

We visualized fluorescently stained samples using either a Hamamatsu MAICO MEMS confocal unit fitted to an inverted microscope (Nikon Eclipse T*i*) or a Crest Optics X‐Light V2tp confocal unit fitted to an inverted microscope (Nikon Eclipse T*i*2) with either 10× air or 20× air objectives. We acquired images using VisiView Software (Visitron Systems) with stage‐stitching and z‐series functions. We post‐processed images, adjusted image contrast and brightness, and made measurements using Fiji.^46^ We calculated lung aspect ratio for each species and developmental stage by measuring proximal‐to‐distal length and the medial‐to‐lateral width of the main bronchus (visualized using immunofluorescence for E‐cadherin). We then divided the length by the width to obtain the aspect ratio.

### 4.5 In silico modeling of epithelial–smooth muscle interactions

We simulated the three‐dimensional morphogenesis of a two‐layered tissue structure using a custom finite element method (FEM) script developed in the Julia programming language, leveraging the Ferrite library.^47^ All simulation code and geometries are publicly available. This simulation follows the methods outlined previously.^16^ Briefly, we treated the processes of growth and pressure as much slower than mechanical relaxation.

Following finite deformation theory, we describe the mapping $x\left(X\right)=X+u\left(X\right)$ from a material point X in the initial reference configuration $\Omega_{0}$ to a point x in the deformed configuration Ω via the displacement field $u\left(X\right)$. The local deformation is characterized by the deformation gradient tensor F=I+∇u. To model growth, we decompose the deformation gradient as $F=F_{e}F_{g}$. Here, $F_{g}$ is a tensor representing isotropic growth, defined as $F_{g}=gI$, where g is the growth factor and I is the identity tensor. The remaining deformation, $F_{e}=FF_{g}^{-1}$, is the elastic deformation that generates mechanical stress.

The tissue layers are modeled as compressible neo‐Hookean hyperelastic materials with a strain energy density function $\psi \left(F_{e}\right)=\frac{\mu }{2}\left(\mathrm{tr}\left(F_{e}^{T}F_{e}\right)-3-2\;\ln J_{e}\right)+\frac{\lambda }{2}{\left(\ln J_{e}\right)}^{2}$, where $\mu \left(X\right)=E\left(X\right)/2\left(1+\nu \left(X\right)\right)$ and $\lambda \left(X\right)=E\left(X\right)\nu \left(X\right)/\left(1+\nu \left(X\right)\right)\left(1-2\nu \left(X\right)\right)$ are the Lamé elastic parameters for material at point X expressed in terms of the Young's modulus $E\left(X\right)$ and Poisson's ratio $\nu \left(X\right)$, and $J_{e}=\det\left(F_{e}\right)$ is the elastic volumetric deformation. This leads to the first Piola–Kirchhoff stress tensor

$$P=\frac{\partial \psi }{\partial F_{e}}=\left(\mu \left(F_{e}-F_{e}^{-T}\;\right)+\lambda \ln\left(J_{e}\;\right)F_{e}^{-T}\;\right).$$

In mechanical equilibrium, the system satisfies equation ∇·P=0 in the bulk domain $\Omega_{0}$ with the boundary condition of the pressure p applied on the inner luminal surface of the epithelium $\partial \Omega_{\text{in}}$ and no tractions applied on the external surface $\partial \Omega_{\text{out}}$. The displacement field u is then found by solving the weak form of the equilibrium for virtual displacements δu:

$$\int_{\Omega_{0}}\;P:\nabla \left(\delta u\right)\mathrm{dV}-\int_{\partial \Omega_{\text{in}}}\;\mathrm{pJ}F^{-T}N\cdot \delta u\mathrm{dS}=0,$$

where we used integration by parts for the first term and used the Nanson's formula for the second term to convert the virtual work of the pressure from the deformed configuration to the undeformed reference configuration, where N is the outward normal in the reference configuration. To ensure a unique solution, we constrained rigid body translation and rotation using Lagrange multipliers.

We discretized the governing nonlinear equations using first‐order tetrahedral elements and solved incrementally using a Newton–Raphson iterative scheme. The simulation linearly ramped the growth factor g and pressure p to their final target values. The model used Young's moduli of *E* = 1.0 and *E* = 10.0 for the epithelium and smooth muscle, respectively, with Poisson's ratio of *ν* = 0.3. Morphogenesis was driven by a total isotropic growth factor of *g* = 2.0 within the epithelium, coupled with a final outward pressure of *p* = −.2 applied to the lumen. Visualizations were generated in ParaView.^48^

## FUNDING INFORMATION

This research was funded, in part, by the Princeton High Meadows Environmental Institute, the National Science Foundation (2134935, 1435853), and the National Institutes of Health (HD099030, HD111539, HL164861, HL166311). AHG and BL were supported by the NSF Postdoctoral Research Fellowships in Biology (PRFB) program (DBI‐2209090 to AHG, DBI‐2305831 to BL). PAT and NAS were supported by the Stowers Institute for Medical Research, and NAS was additionally supported by a K99/R00 Pathway to Independence Award from the NICHD (HD114881).

## CONFLICT OF INTEREST STATEMENT

The authors of this study declare no conflicts of interest.

## Supporting information


**Data S1.** Supporting Information.

## Data Availability

The data that support the findings of this study are available on request from the corresponding author. The data are not publicly available due to privacy or ethical restrictions.
