# Supplementary material for: Biophysical processes of morphogenesis in lizard lungs
Source: Dev Dyn. 2025 Nov 14;255(9):867–80. doi: 10.1002/dvdy.70097 (PMC13553195; doi:10.1002/dvdy.70097)
Supplement: Supplementary file 1 [file DVDY-255-867-s001.docx]

**Supplemental Figures**

**Supplemental Figure 1. Opportunistic sampling, developmental staging, and aspect ratios of Italian wall lizard lungs.** Images show immunofluorescence for E-cadherin and aSMA (**A**–**D**). St, developmental stage, Scale bars, 300 µm. White arrow denotes emergence of the proximal sub-chamber. Gray arrows denote epithelium pushing through the smooth muscle mesh. **E**) A photograph of an Italian wall lizard—a lizard with a single-chambered lung.

**Supplemental Figure 2. Schematic depicting procedure used to dissect lizard lungs**. **A**) Illustration of a lizard embryo in lateral view. Dashed box corresponds to the region of the torso where lungs reside. **B**) Illustration of the lateral view of lizard viscera. **C**) Illustration of a lizard embryo in lateral view depicting regions to cut with dissection forceps (red lines). **D**) Illustrations of lizard viscera in dorsal view. The lung is visible after removal of the mesonephros.

**Supplemental Table 1.** **Average aspect ratios for individual lizard lungs.**

| species | embryonic stage | aspect ratio |
| --- | --- | --- |
| brown anole | 32 | 3.50 |
| brown anole | 32 | 3.67 |
| brown anole | 32 | 2.96 |
| brown anole | 33 | 1.86 |
| brown anole | 33 | 2.41 |
| brown anole | 33 | 2.18 |
| brown anole | 35 | 1.64 |
| brown anole | 35 | 2.25 |
| brown anole | 35 | 2.30 |
| leopard gecko | 32 | 3.23 |
| leopard gecko | 32 | 2.60 |
| leopard gecko | 32 | 3.36 |
| leopard gecko | 33 | 2.56 |
| leopard gecko | 33 | 2.75 |
| leopard gecko | 33 | 2.79 |
| leopard gecko | 36 | 3.50 |
| leopard gecko | 36 | 3.21 |
| leopard gecko | 36 | 2.25 |
| veiled chameleon | 32 | 7.11 |
| veiled chameleon | 32 | 4.12 |
| veiled chameleon | 32 | 7.10 |
| veiled chameleon | 33 | 4.05 |
| veiled chameleon | 33 | 6.82 |
| veiled chameleon | 33 | 5.36 |
| veiled chameleon | 35 | 4.36 |
| veiled chameleon | 35 | 5.32 |
| veiled chameleon | 35 | 3.79 |
| Italian wall lizard | 33 | 2.93 |
| Italian wall lizard | 34 | 3.28 |
| Italian wall lizard | 35 | 4.46 |
| Italian wall lizard | 37 | 2.39 |
